# Proteome Analysis of Thyroid Hormone Transporter Mct8/Oatp1c1-Deficient Mice Reveals Novel Dysregulated Target Molecules Involved in Locomotor Function

**DOI:** 10.3390/cells12202487

**Published:** 2023-10-19

**Authors:** Devon Siemes, Pieter Vancamp, Boyka Markova, Philippa Spangenberg, Olga Shevchuk, Bente Siebels, Hartmut Schlüter, Steffen Mayerl, Heike Heuer, Daniel Robert Engel

**Affiliations:** 1 Department of Immunodynamics, Institute for Experimental Immunology and Imaging, University Duisburg-Essen, 45141 Essen, Germany; devon.siemes@uk-essen.de (D.S.); philippa.spangenberg@uni-due.de (P.S.); olga.shevchuk@uk-essen.de (O.S.); engel@immunodynamics.de (D.R.E.); 2 Department of Endocrinology, Diabetes and Metabolism, University Hospital Essen, University Duisburg-Essen, 45147 Essen, Germany; pieter.vancamp@univ-nantes.fr (P.V.); boyka.markova@uk-essen.de (B.M.); steffen.mayerl@uk-essen.de (S.M.); 3 Section Mass Spectrometric Proteomics, Diagnostic Center, University Medical Center Hamburg-Eppendorf, 20246 Hamburg, Germany; b.siebels@uke.de (B.S.); hschluet@uke.de (H.S.)

**Keywords:** T4, T3, Slc16a2, Slco1c1, CNS, myelination, basal ganglia, Mct8, Oatp1c1, Allan–Herndon–Dudley Syndrome

## Abstract

Thyroid hormone (TH) transporter MCT8 deficiency causes severe locomotor disabilities likely due to insufficient TH transport across brain barriers and, consequently, compromised neural TH action. As an established animal model for this disease, Mct8/Oatp1c1 double knockout (DKO) mice exhibit strong central TH deprivation, locomotor impairments and similar histo-morphological features as seen in MCT8 patients. The pathways that cause these neuro-motor symptoms are poorly understood. In this paper, we performed proteome analysis of brain sections comprising cortical and striatal areas of 21-day-old WT and DKO mice. We detected over 2900 proteins by liquid chromatography mass spectrometry, 67 of which were significantly different between the genotypes. The comparison of the proteomic and published RNA-sequencing data showed a significant overlap between alterations in both datasets. In line with previous observations, DKO animals exhibited decreased myelin-associated protein expression and altered protein levels of well-established neuronal TH-regulated targets. As one intriguing new candidate, we unraveled and confirmed the reduced protein and mRNA expression of Pde10a, a striatal enzyme critically involved in dopamine receptor signaling, in DKO mice. As altered PDE10A activities are linked to dystonia, reduced basal ganglia PDE10A expression may represent a key pathogenic pathway underlying human MCT8 deficiency.

## 1. Introduction

Thyroid hormone (TH) transport across cellular membranes is a key step in TH signaling as TH metabolism and action occur intracellularly. The monocarboxylate transporter 8 (MCT8) encoded by the X-linked *SLC16A2* gene represents a highly specific TH transporter for both the prohormone T4 (3,3′,5,5′-tetraiodothyronine; thyroxine) as well as the more receptor active form T3 (3,3′,5-triiodothyronine) [1]. Inactivating mutations in MCT8 in humans cause peripheral thyrotoxicity and severe neurocognitive and motor impairments with hypotonia and hypokinesis as key clinical features, better known as Allan–Herndon–Dudley Syndrome (AHDS) [2,3,4]. These neuro-motor symptoms are considered to be due to an impaired TH transport across brain barriers and further into neural cells that, consequently, compromises proper brain development and function [5]. 

Mice with a concomitant inactivation of Mct8 and the T4-specific transporter Oatp1c1 (Mct8/Oatp1c1 knockout (DKO) mice) replicate the peripheral endocrine abnormalities of MCT8 patients and show a profound TH-deficient status in the CNS, thus representing an established animal model of AHDS [6]. DKO mice also display locomotor impairments as well as distinct histo-morphological alterations, such as impaired cortical GABAergic cell differentiation, delayed Purkinje cell dendritogenesis and persistent hypomyelination [6,7,8,9]. These observations align with results from MRI analyses and post-mortem brain samples derived from a MCT8-deficient patient [10,11,12]. 

Similar morphological anomalies are frequently seen in mouse models with impaired central TH signaling due to genetic manipulations or anti-thyroid drug treatment [13,14,15]. Yet, the RNA sequencing of cerebral and striatal tissues of postnatal DKO mice revealed a significant number of dysregulated genes that do not overlap with those seen in mice, which were rendered systemically hypothyroid by an anti-thyroid drug treatment [16]. Likewise, the clinical profile of AHDS does not fully recapitulate that of patients with neurological cretinism, which is caused by a lack of TH supply during prenatal stages [17]. It is therefore reasonable to assume that specific brain regions and/or cell types rely, to a different extent, on MCT8-mediated TH transport and that other, yet unknown TH transporters may be able to compensate for the absence of MCT8 in certain cell types and developmental stages [5]. Finally, it also cannot be ruled out that, in addition to TH, MCT8 may transport additional compounds that are essential for normal neuronal differentiation and function in distinct brain areas and that directly or indirectly influence the expression of CNS genes independent of TH.

We therefore pursued to assess alterations in the proteomic landscape in DKO mice by an unbiased approach using mass spectrometry-based proteomics on brain sections of DKO mice and wildtype (WT) controls. In order to compare possible changes in the proteomic landscape with those recently found in transcriptome analyses [16], we studied mice at the same age (at postnatal day P21) and focused on brain sections largely comprising cortical and striatal areas. By bioinformatic analysis, we were not only able to confirm and expand the list of proteins linked to deficits in myelination and neurogenesis, but also disclosed distinct alterations in signaling pathways and in the expression of specific molecules that have not been considered as targets of TH action in AHDS, but that in humans are linked to movement disorders and may thus be of relevance for patients with MCT8 deficiency.

## 2. Methods

### 2.1. Animals

WT and DKO mice on a C57BL/6 background were generated and genotyped as published elsewhere [6]. Mice were kept at a constant temperature (22 °C) on a 12 h light, 12 h dark cycle and were provided with standard laboratory chow and water ad libitum. For proteome analyses, male mice at postnatal day 21 (P21) were deeply anaesthetized by ketamine/xylazine application and subjected to terminal perfusion fixation using a 4% PFA solution. Brains were cryo-protected in 30% sucrose in PBS, frozen in isopentane on dry ice and stored at −80 °C. For mass spectrometry-based proteomics, 20 μm thick coronal brain sections between Bregma 0.98 and 0.86 mm were produced on a cryostat and kept as single sections in 1.5 mL Eppendorf tubes. Subsequently, sections for immuno-fluorescence analyses were cut between Bregma 0.86 and 0.62 mm from the same brains. For fluorescence in situ hybridization (FISH) experiments, P21 (male and female) and P120 (male only) mice were killed by cervical dislocation, brains were frozen in isopentane on dry ice and used to prepare 20 μm thick cryosections from the same Bregma area as above. For Western blotting, the forebrains of P21 female mice were bisected along the midline and each hemisphere was snap-frozen on dry ice. All samples were stored at −80 °C until further processing.

### 2.2. Protein Extraction and Tryptic Digestion

PFA-fixed brain samples were dissolved in 100 mM triethyl ammonium bicarbonate (TEAB) and 1% *w*/*v* sodium deoxycholate (SDC) and boiled at 95 °C for 1 h for reverse formalin fixation and protein denaturation. Sonication was performed for 6 pulses at 35 Hz to eliminate DNA and RNA. The protein concentration was determined by the Pierce TM BCA Protein assay kit following the manufacturer’s instructions. A total of 20 μg of protein for each sample were used for tryptic digestion. Disulfide bonds were reduced in 10 mM dithiothreitol for 30 min, alkylated in presence of 20 mM iodoacetamide for 30 min in the dark and digested with trypsin (sequencing grade, Promega) at 1:100 (enzyme:protein) and 37 °C overnight. SDC was precipitated by 1% *v*/*v* formic acid followed by centrifugation at 16,000× *g*. The supernatant was dried in a vacuum centrifuge and stored at −20 °C until further use. 

### 2.3. High pH Fractionation

Thirteen high-pH reversed-phase chromatography fractions were used for spectral library generation. For fractionation, equal amounts of all analyzed samples were dissolved in 10 mM NH_4_HCO_3_ to a final concentration of 1 μg/μL. In total, 50 μg of digested proteins were used for high-pH RP-HPLC using a 25 cm ProSwift™ RP-4H capillary monolithic column (Thermo Fisher, Waltham, MA, USA) on an Agilent 1200 series HPLC (high-pressure liquid chromatography) system. A gradient was applied for a total of 45 min with a flow rate of 0.2 mL/min, starting at 96.7% eluent A (10 mM NH_4_HCO_3_) and 3.3% eluent B (10 mM NH_4_HCO_3_ in 90% acetonitrile (ACN)) for 5 min, rising to 38.5% B in 20 min, increasing to 95.0% in 1 min for 10 min and re-equilibrated to 3.3% B for 8 min. A total of 30 fractions were collected on an Äkta prime plus fraction collector, pooled to 13 fractions and dried in the vacuum centrifuge.

### 2.4. LC-MS/MS Acquisition

Prior to liquid-chromatography-coupled tandem mass spectrometry (LC-MS/MS) analysis, samples were dissolved in 0.1% formic acid (FA) to a final concentration of 1 μg/μL. For LC-MS/MS, 1 μg of tryptic peptides were injected for individual samples and library fractions alike. The measurements were performed on a quadrupole-ion-trap-orbitrap MS (Orbitrap Fusion, Thermo Fisher) coupled to a nano-UPLC (Dionex Ultimate 3000 UPLC system, Thermo Fisher). The chromatographic separation of peptides was achieved with a two-buffer system (buffer A: 0.1% FA in water, buffer B: 0.1% FA in ACN). Attached to the UPLC was a peptide trap (100 μm × 200 mm, 100 Å pore size, 5 μm particle size, C18, Thermo Fisher) for online desalting and purification followed by a 25 cm C18 reversed-phase column (75 μm × 250 mm, 130 Å pore size, 1.7 μm particle size, Peptide BEH C18, Waters, Milford, MA, USA). The peptides were separated using an 80 min gradient with linearly increasing ACN concentration from 2% to 30% ACN in 65 min. Eluted peptides were ionized using a nano-electrospray ionization source (nano-ESI) with a spray voltage of 1800 V, transferred into the MS and analyzed in the data-dependent acquisition (DDA) mode. For each MS1 scan, ions were accumulated for a maximum of 120 ms or until a charge density of 2 × 10^5^ ions (AGC Target) was reached. The Fourier-transformation-based mass analysis of the data from the orbitrap mass analyzer was performed covering a mass range of 400–1300 *m*/*z* with a resolution of 120,000 at *m*/*z* = 200. Peptides with charge states in the range of 2–5+, above an intensity threshold of 1000, were isolated within a 1.6 *m*/*z* isolation window in the Top Speed mode for 3 s from each precursor scan and fragmented with a normalized collision energy of 30% using higher energy collisional dissociation (HCD). MS2 scanning was performed using an ion trap mass analyzer at a rapid scan rate, covering a mass range of 380–1500 *m*/*z* and accumulated for 60 ms or to an AGC target of 1 × 10^5^ ions. The already fragmented peptides were excluded for 15 s. 

### 2.5. Data Processing 

LC-MS/MS data were searched with the Sequest algorithm integrated in the Proteome Discoverer software (v 2.4.1.15, Thermo Fisher) against a reviewed murine Swissprot database, obtained in October 2020, containing 17,053 entries. Carbamidomethylation was set as a fixed modification for cysteine residues, and the oxidation of methionine, pyro-glutamate formation at glutamine residues at the peptide N-terminus as well as the acetylation of the protein N-terminus were allowed as variable modifications. A maximum number of 2 missing tryptic cleavages was set. Peptides between 6 and 144 amino acids were considered. A strict cutoff (FDR < 0.01) was set for peptide and protein identification. Quantification was performed using the Minora Algorithm, implemented in Proteome Discoverer. LC-MS/MS from high-pH fractions were searched, like the single samples, by adding the 13 fractions as a single file set. Afterwards, both result files were used to create a consensus and processed in a consensus workflow. Subsequently, the results were filtered for high confident peptides, with enhanced peptide and protein annotations. Protein abundances for individual samples were exported for subsequent statistical analysis.

### 2.6. Transcriptomic Data

Published data on DKO transcriptome were used [16] and the striatal transcript dataset was compared with the proteomic dataset generated in this study. 

### 2.7. Western Blot

Fresh-frozen forebrain hemispheres were lysed in RIPA-buffer (150 mM NaCl, 50 mM HCl, 1% Nonidet P-40, 0.5% sodium deoxycholate, 0.1% SDS, 2 mM EDTA and 50 mM NaF; 5 mL for approximately 0.5 g of tissue), to which half a tablet of cOmplete^TM^ protease inhibitor cocktail (Roche, Basel, Switzerland) was added. The tissue was homogenized using ceramic beads and the Minilys system (Bertin technologies, Montigny-Le-Bretonneux, France) at 5000 rpm for 30 s, and put on ice for 20 min prior to centrifugation at 14,000× rpm for 20 min at 4 °C. Protein concentrations were determined using the BCA method according to the manufacturer’s instructions (Thermo Fisher). Then, 2× Laemmli buffer (BioRad, Hercules, CA, USA) was added to 20 μg of protein per sample, vortexed for 5 s, put on ice and not heated to prevent membrane protein aggregation. Samples were loaded and separated on a 12% SDS-polyacrylamide gel for SDS-PAGE at 80 V for 2 h. Proteins were transferred onto a PVDF membrane at 15 V and 4 °C for 16 h and blocked in 5% *w*/*v* non-fat milk powder in TBST for 1 h, followed by incubation with primary antibodies (rabbit-anti-Pde10a, 1:1000, ab227289, Abcam, Cambridge, UK; rabbit-anti-Gapdh, 1:1000, 2118L, Cell Signalling, Danvers, MA, USA) overnight at 4 °C in blocking buffer. Blots were washed 3× in TBST and incubated with an HRP-conjugated goat-anti-rabbit secondary antibody (1:2000, Cell Signalling) for 1 h at room temperature. Following three washes with TBST, blots were incubated with 500 μL ECL reagent (1/1, BioRad, 170-5060) and signals were visualized using a VersaDoc MP 400 system (BioRad). 

### 2.8. Immunohistochemistry

Sections were washed in PBS + 0.2% Triton-X100 (PBST) for 10 min and microwave-heated for 10 min in sodium citrate buffer (10 mM sodium citrate, 0.05% Tween; pH 6.0) to retrieve the antigens. Subsequently, the sections were blocked with 10% donkey serum in PBST, after which primary antibodies (rabbit-anti-Pde10a, 1:1000, ab227289, Abcam; mouse-anti-Parvalbumin (Pvalb), 1:500, MAB1572, Millipore, Burlington, MA, USA) were applied overnight at 4 °C. The slides were washed and incubated with Alexa488-conjugated donkey-anti-rabbit and Alexa55-conjugated donkey-anti-mouse secondary antibodies (1:1000, Thermo Fisher) for 2 h at room temperature. After 10 min incubation with Hoechst33258 (1:10,000, Invitrogen, Waltham, MA, USA), the slides were covered with Fluoromount^TM^ Aqueous Mounting Medium (Sigma, St. Louis, MO, USA) and cover-slipped. Pictures from the dorsal striatum on two sections per brain were taken using a SP8 confocal microscope (Leica, Wetzlar, Germany) (for Pde10a) or a Zeiss Apotome (for Pvalb). 

### 2.9. Fluorescence In Situ Hybridization (FISH) 

Fresh-frozen cryosections were pretreated as described elsewhere [18]. In brief, frozen coronal forebrain sections (20 μm) were air-dried, fixed in a 4% PFA solution (pH 7.4) for 60 min and permeabilized in 0.4% Triton-X 100 containing PBS for 10 min. The sections were dehydrated and covered with hybridization mix. Third-generation FISH experiments were performed as described elsewhere [19]. Probes against Pde10a consisting of a set of 20 split-initiator probe pairs and coupled to the probe-specific HCR initiator B5 for the target were commercially designed and generated. Probes, buffers and fluorescently labeled hairpins were purchased from Molecular Instruments, Los Angeles, CA, USA. Hybridization buffer was applied for 10 min at 37 °C before probes in hybridization buffer (0.4 pmol per 100 μL) were added to the sections and hybridization was conducted for 24 h at 37 °C. The sections were washed with a probe wash buffer and 5× SSC + 0.1% Tween20 (SSCT) and incubated with amplification buffer for 30 min at room temperature. B5-specific hairpins h1 and h2 labelled with Alexa488 fluorophore (6 pmol per 100 μL amplification buffer) were separately heat-shocked for 90 s at 95 °C and cooled down to room temperature for 30 min. Hairpins were mixed in amplification buffer and applied onto the slides. Signal amplification was conducted for 16 h at room temperature. The slides were rinsed in SSCT, incubated for 5 min with Hoechst33258 (1:10,000, Invitrogen), rinsed in SSCT, cover-slipped and analyzed using a SP8 confocal microscope (Leica).

### 2.10. Data Analysis, Statistics and Visualization

Proteomic data were normalized using loess normalization (R package) and subsequently imputed using a uniform random 5th–10th percentile strategy. The statistical significance was tested by a Welch *t*-test and the resulting *p*-values were corrected for false discovery rate (FDR) using the Benjamini–Hochberg procedure (q-value). Signal-to-noise-ratio (SNR) was calculated as $SNR=\frac{\bar{x_{1}}-\bar{x_{2}}}{d_{1}+d_{2}}$, where $\bar{x_{1}}$ and $\bar{x_{2}}$ are the mean and $d_{1}$ and $d_{2}$ are the standard deviation of the conditions. The log_2_-fold change (log_2_FC) was determined as $\log_{2}\;FC=log_{2}\left(\frac{\bar{x_{1}}}{\bar{x_{2}}}\right)$. Heatmap, violin, box, regression scatter and volcano plots, clustermap and principal component analysis (PCA) were generated using the Python package matplotlib and seaborn. The overrepresentation analysis (ORA) for analyzing functional enrichments was performed using terms from the Molecular Signatures Database (MSigDB) category M5-GO “Gene Ontology gene sets” (m5.go.v2023.1.Mm.entrez.gmt) and clusterprofiler (v4.6.0), considering molecules with a q-value < 0.01. All measured proteins were included in the background distribution and resulting *p*-values were corrected for FDR with the Benjamini–Hochberg procedure to determine the q-value. Cytoscape v3.9.1 was used for generating the string network to map differentially expressed genes retrieved from the proteomics analysis. Using the interface, the top 50 genes linked to the term “dystonia” for *Mus musculus* (Full String Network, with a confidence score of 0.70) were retrieved from PubMed, after which the differentially expressed genes/proteins were mapped on this string network and colored according to their relative expression.

For FISH experiments, mean grey values of the fluorescent signals were measured in striatal areas using ImageJ. Likewise, Pde10a protein levels were determined by encircling striatal areas and measuring the mean grey values of the fluorescent signals using ImageJ. The number of Pvalb positive cells was counted in the striatum and normalized to the area size. Three to four pictures per animal were analyzed. For the Western blot analysis, integrated densities of the bands were measured on 8-bit converted images using ImageJ and normalized to Gapdh as a housekeeping control. 

All data represent mean ± SD. An unpaired Student’s *t*-test was performed using GraphPad Prism 5. Differences were considered significant when *p* < 0.05 and were marked as follows: * *p* < 0.05; ** *p* < 0.01; *** *p* < 0.001. 

## 3. Results

### 3.1. Mass-Spectrometry-Based Proteomic Analysis 

**Figure 1:**
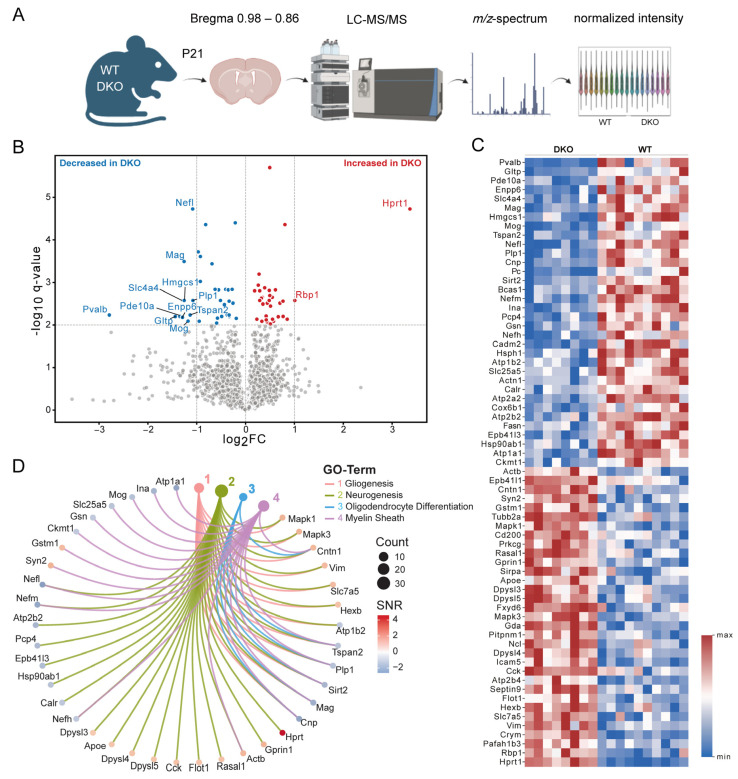
Proteomic profiling of brain sections by LC-MS/MS. (**A**) Brain sections of WT and DKO mice at P21 were analyzed by LC-MS/MS. (**B**) Volcano plot displaying the regulation of proteins indicated by −log_10_ q-value and log_2_FC. Proteins with q < 0.01 were colored red and blue to indicate significant up- and down-regulation, respectively, in DKO versus WT animals. Grey dots indicate proteins that were not found to be differentially expressed. (**C**) Heatmap of the 67 regulated proteins with a q < 0.01 sorted by descending fold change. (**D**) Spider plot of the top 4 non-redundant, enriched pathways and their annotated genes from the over-representation analysis with the mouse-specific MSigDB category M5-GO “Gene Ontology gene sets” using the significantly regulated proteins. SNR: signal-to-noise ratio. n = 10 (WT) and n = 8 (DKO).

To study the changes in the proteomic landscape in Mct8/Oatp1c1-deficient mice, we employed liquid chromatography-tandem mass spectrometry (LC-MS/MS) on coronal brain sections of P21 mice between Bregma 0.98 and 0.86 mm, hence comprising largely cortical and striatal areas (Figure 1A). For the pre-processing of the LC-MS/MS data, the protein abundance distributions of all samples after loess normalization and imputation were determined (Supplementary Figure S1A). Principal component analysis (Supplementary Figure S1B) and Pearson correlation clustermap (Supplementary Figure S1C) with proteins filtered by FDR-corrected *p*-value (q-value) < 0.01 indicated the clustering of the samples of both conditions without outliers. The visualization of the q-values and log2-fold changes (log_2_FC) among the WT and DKO mice indicated 67 out of 2913 significantly (q < 0.01) regulated proteins (Figure 1B and Supplementary Table S1), of which 33 were significantly upregulated and 34 were significantly downregulated in DKO mice. Ten proteins had a log_2_FC < −1 and two a log_2_FC > +1. We generated a heatmap to plot the expression levels of the dysregulated proteins according to descending log_2_FC for each sample in both conditions (Figure 1C). Those proteins were used for over-representation analysis using the mouse-specific Molecular Signature Database (MSigDB) with the category M5-Gene Ontology (GO) gene sets. Using this analysis, four non-redundant, enriched terms with a q value < 0.05, which were associated with neurogenesis, gliogenesis, myelin sheath and oligodendrocyte differentiation, were annotated (Figure 1D). 

### 3.2. Comparison between Transcriptomic and Proteomic Data

**Figure 2:**
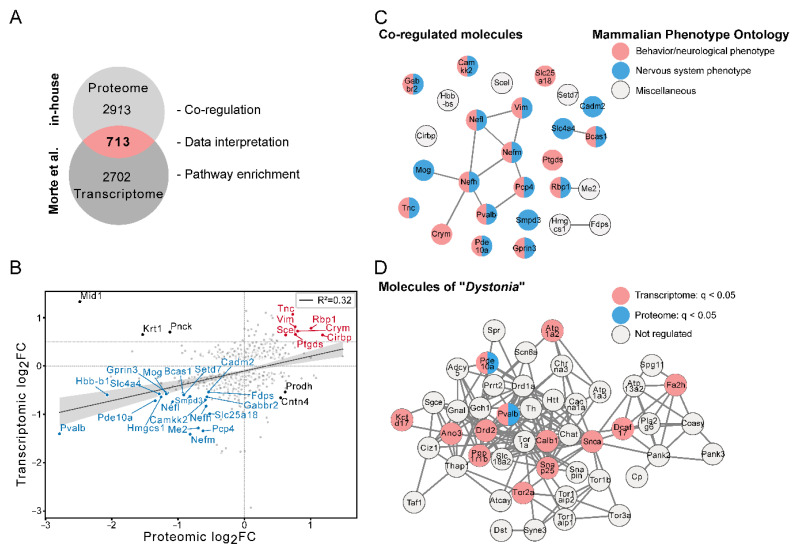
Comparison of proteomic and transcriptomic alterations. (**A**) Venn diagram indicating overlapping molecules between our P21 DKO proteome data and a published transcriptomic dataset [16] on age-matched DKO striatum. (**B**) Regression scatter plot of the 713 overlapping molecules based on the interception of the Venn diagrams with their respective proteomic and transcriptomic log_2_FC. Molecules that are down-regulated on the transcript and protein level are depicted in blue, while those that are up-regulated in tandem are shown in red. Black dots indicate molecules that are differentially expressed on the transcript and protein level with opposite directions. Factors that are not concomitantly regulated in both datasets are displayed in grey. The grey band along the regression line indicates the regression’s 95% confidence interval. (**C**) StringDB analysis of co-regulated molecules with a log_2_FC for transcriptome and proteome data >0.5. The molecules labelled in grey are not annotated to any of the highlighted terms and were therefore collectively grouped in the term “miscellaneous”. (**D**) The top 50 molecules of the term dystonia, according to PubMed datamining, were compared to the proteomic and transcriptomic regulations in DKO versus WT mice, revealing Pde10a and Pvalb as key affected molecules.

To elucidate which of these molecules were co-regulated in DKO mice on transcriptomic and proteomic levels, we used a recently published bulk RNA sequencing-generated transcriptome dataset of the striatum and cerebral cortex of WT and DKO animals at the same age (P21) [16]. The intersection analysis revealed an overlap of 713 molecules with the striatal transcriptome dataset (Figure 2A and Supplementary Table S1), all of which were used for further data interpretation, co-regulation assessment and pathway enrichment. The regression scatter plot of the log_2_FC of our in-house proteomic and the recently published transcriptomic dataset indicated a positive linear correlation between the expression levels of mRNAs and proteins (Figure 2B). Among the 713 common molecules, 27 showed a similarly altered regulation in both transcriptomic and proteomic data, i.e., increased mRNA levels correlated with a higher protein abundance (7 molecules) and vice-versa (20 molecules). As one example, the intracellular TH-binding protein μ-Crystallin/Crym was up-regulated on the transcript and protein level as expected from previous studies [6]. Interestingly, five molecules (Mid1, Krt1, Pnck, Prodh and Cntn4) revealed opposing expression patterns between the datasets (Figure 2B). The 27 co-regulated molecules that were altered in both datasets were then subjected to “Mammalian Phenotype Ontology” analysis using STRING (Search Tool for the Retrieval of Interacting Genes/Proteins). Interestingly, 20 of these factors were associated with the terms “Behavioral/neurological phenotype” and “Nervous system phenotype” (Figure 2C). Based on these terms, we wondered whether some of the 20 identified factors may be involved in the neuro-motor phenotype that is a key neurological feature of AHDS [11,20]. We therefore conducted a datamining approach using PubMed search and extracted the top 50 candidates that are linked to the term “Dystonia”. Using STRING network analysis, we observed that several of those molecules were differentially expressed in DKO mice (Figure 2D). Of note, Pvalb and Pde10a were down-regulated at the transcriptomic and proteomic levels.

### 3.3. Candidate Molecule Analysis in the Striatum

The calcium-binding protein Pvalb labels a specific subset of inhibitory GABAergic interneurons and constitutes a classical marker for proper TH signaling during brain development [21]. Its downregulation in both AHDS patients and DKO mice is well-documented in the cerebral cortex [6,7,10]. To validate our finding of insufficient expression in the striatum, we subjected coronal forebrain sections from P21 WT and DKO male mice to Pvalb immuno-staining (Supplementary Figure S2). In agreement with previous reports, we demonstrated a reduced number of Pvalb+ interneurons in the DKO striatum, pointing to impaired inhibitory GABAergic neurotransmission. 

**Figure 3:**
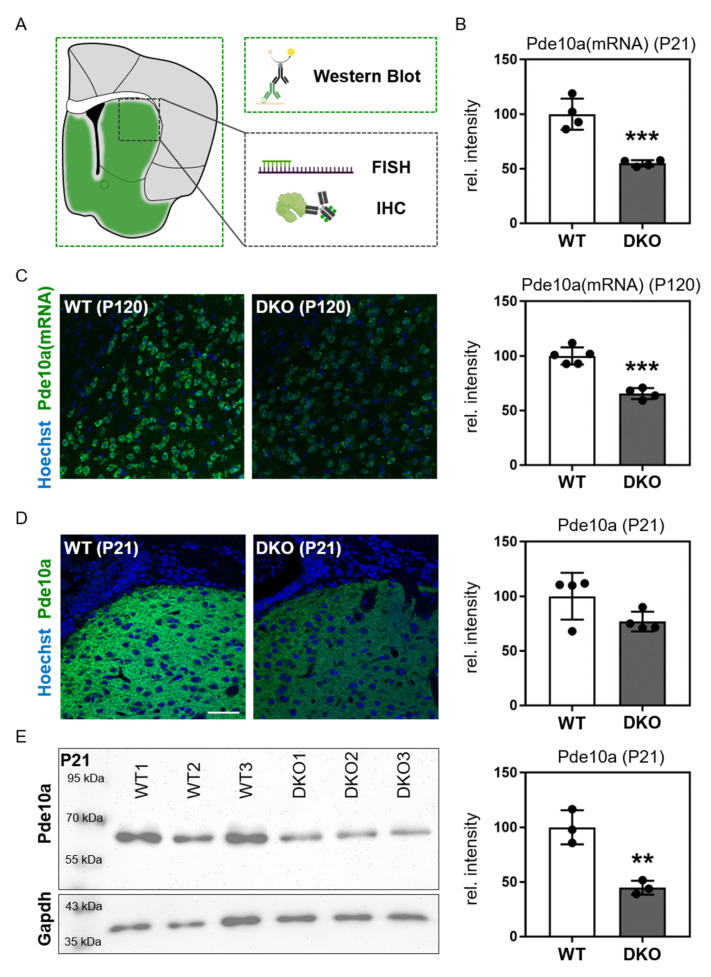
Reduced Pde10a mRNA and protein levels in the striatum of DKO mice. (**A**) Illustration of the analyzed brain areas for FISH and IHC (striatum; black box), and Western blot (whole forebrain hemisphere; green box). Icons were created with BioRender.com (accessed on 2 September 2023). (**B**,**C**) Fluorescence in situ hybridization analysis of striatal *Pde10a* mRNA (in green) expression revealed significantly reduced signal intensities in DKO mice at P21 ((**B**) WT: 3 females and 1 male; DKO: 2 females and 2 males) and P120 ((**C**) all males). (**D**) Immunofluorescence staining for Pde10a protein (in green) demonstrating reduced signal intensities in DKO mice at P21. (**E**) Western blot analysis of forebrain homogenates confirmed reduced Pde10a protein levels in female DKO mice at P21. As a housekeeping protein for the normalization of signal intensities, Gadph was used. Blue color in (**C**,**D**) indicates Hoechst33258-counterstained cell nuclei. Scale bar: 50 μm. n = 3–5. ** *p* < 0.01; *** *p* < 0.001.

The second dystonia-related candidate that we identified in our bioinformatic assessments was Pde10a, a cAMP- and cGMP-inactivating phosphodiesterase that is specifically expressed in striatal GABAergic medium spiny neurons (MSNs) and that regulates dopamine receptor signaling [22]. As Pde10a has not yet been studied in the context of TH-regulated striatal function, we aimed to validate our finding and examined Pde10a expression both at the transcript and protein levels in WT and DKO mice (Figure 3A). Using Pde10a-specific probes, we conducted FISH analysis on coronal forebrain sections at postnatal days P21 (Figure 3B) and P120 (Figure 3C). Pde10a mRNA specific hybridization signals were found only in striatal areas, whereas the cerebral cortex was devoid of any hybridization signal. Quantifying hybridization signal intensities in the striatum revealed an almost 50% downregulation of Pde10a mRNA expression in DKO mice at both time points (Figure 3B,C). Immuno-fluorescence staining and Western blot analysis using a Pde10a-specific antibody confirmed the reduced expression of Pde10a protein in DKO mice (Figure 3D,E, respectively). 

In conclusion, our study revealed global changes in the proteomic landscape of DKO mice and disclosed Pde10a as a novel striatal TH target molecule. Its dysregulated expression in the striatum suggests a role in the disturbed basal ganglia circuit activity in DKO mice.

## 4. Discussion

Patients with MCT8 deficiency exhibit severe cognitive and locomotor disabilities that are thought to be caused by a diminished TH uptake across brain barriers and into neural cells [5]. As the most consistent and prominent clinical feature, patients display hypokinesis and hypotonia with a poor head control that often progress to spasticity and/or dystonia, suggesting that basal ganglia circuit function might be disturbed [11,20,23]. As another frequently observed clinical finding, abnormal myelination has been reported in several MRI studies of AHDS patients and confirmed in post-mortem histo-morphological studies [10,11,24], which may contribute to the motor dysfunction. Likewise, mice deficient for the TH transporters Mct8 and Oatp1c1 (DKO) replicate many clinical features of human MCT8 deficiency. DKO mice show locomotor impairments, abnormal GABAergic interneuron development as well as a persistent hypomyelination due to insufficient TH supply to neurons and oligodendrocytes [6,9]. However, to which extent neuronal and myelination deficits cause the observed locomotor impairments in DKO mice remains a matter of debate. Similarly elusive is the question as to the exact pathogenic pathways that underlie this neuro-developmental disorder.

In this paper, we conducted an unbiased proteomic profiling of forebrain sections by LC-MS/MS in order to obtain new insights into putatively altered neural circuits in DKO mice. To that end, we employed three-week-old animals as we characterized their thyroidal state in depth in previous studies [6]. Moreover, we focused on cortical and striatal areas, which enabled us to compare our results directly with recently published transcriptome data [16]. In our study, we provided the first LC-MS/MS-based proteomic dataset of an AHDS mouse model with more than 2900 detected proteins, which will serve as a valuable, publicly available database for future analyses.

Our bioinformatic analysis revealed changes in protein expression patterns in DKO mice that are in line with previous findings. Gene ontology assessment disclosed alterations in neurogenesis- and myelination-related factors as major features in DKO animals, both highlighting their pathological impact and emphasizing the validity of our analyses. Of the 67 identified candidates that exhibited a significantly altered protein abundance in DKO mice, 33 proteins were up- and 34 were downregulated. A prominent observation is the downregulation of several myelin-associated proteins (Mog, Mag, Plp1 and Cnp), which fully reflects the myelination phenotype of DKO mice and AHDS patients [6,10]. Likewise, a reduced abundance of proteins known to be present within the oligodendroglia lineage (Bcas, Enpp6, Gltp, Sirt2 and Smpd3) is in agreement with a decreased number of oligodendrocytes in white and grey matter regions of DKO mice [9]. The decreased protein expression of several neurofilaments (Nefl, Nefm, Nefh and Ina1) are indicative for a disturbed neuronal maturation and might be linked to the reduced axon caliber observed in MCT8 patients and AHDS mouse models [6,12,25]. Moreover, we identified several proteins in the DKO proteome that showed increased abundance and are potentially involved in synaptic plasticity (Gda, Syn2 and Gprin1). The implied altered synaptic transmission aligns with our recent finding of a brain-wide hyper-connectivity in DKO mice [8], although it remains elusive whether this is solely due to the impaired development of the inhibitory GABAergic system in that mouse model or whether other neurotransmitter systems are affected as well. 

Of the 67 proteins that are differentially expressed in DKO mice, 32 were also regulated at the mRNA level, as demonstrated by our comparison with a recently published striatal transcriptomic dataset [16]. Most of those molecules (27 out of 32; 85%) were regulated in the same direction, both at the transcript and protein levels. Of those, 16 have been shown to exhibit a thyroid hormone receptor-binding site (TRBS) and may therefore constitute direct targets of TH receptor action [26]. The relatively low number of molecules is contrasted with the numerous molecules that are found in both datasets, but only significantly regulated at the transcript or proteome level, or that are even only detected by one analytical approach or the other. This obvious discrepancy may originate from the comparison of a pure striatal transcriptomic dataset with our proteomic dataset that encompasses largely cortical and striatal areas, but also contains corpus callosum and septal regions and thus might mitigate some area-specific effects. However, despite these pitfalls, the obvious differences between transcriptomic and proteomic alterations council caution regarding the interpretation of transcript data and their direct translatability towards functional aspects. To that end, proteome analyses represent a much more powerful tool that has just started to be implemented in complexes diseases, such as AHDS, and that will gain even more impact with the current rapid improvements in spatial proteome imaging techniques. 

Particularly interesting to us were those factors that are differentially expressed both in the transcriptomic and proteomic profiling, but which were not previously reported to be altered in Mct8/Oatp1c1 deficiency and may thus advance our understanding of the yet elusive pathways underlying the pathological alterations in AHDS. Noteworthy candidates amongst them are Cadm2 (cell adhesion molecule 2), which participates in axon pathfinding and axon–axon contacts, and Smpd3 (neutral sphingomyelinase 2), which is involved in lipid metabolism and is a known T3 target gene in the striatum [27,28,29]. A third molecule that is significantly reduced in DKO mice at both the transcriptomic and proteomic levels is Pde10a, a phosphodiesterase that inactivates both cAMP and cGMP [30]. That this decrease is seen both at P21 and P120 as well as in male and female mice underscores the robustness of the effect and argues against a sexual dimorphism. This latter finding may have important implications as our STRING network analysis highlighted Pde10a, together with the known TH-sensitive inhibitory interneuron marker Pvalb, in a network of factors associated with dystonia and may thus be involved in the dysfunctional locomotor activity in AHDS patients and DKO mice. 

In both humans and mice, Pde10a is highly and almost exclusively expressed in striatal MSNs, which function as a gateway for the processing of cortical information to the basal ganglia circuitry, thereby enabling the dynamic integration of cortical information into locomotor and behavioral actions [31,32,33,34]. Owing to its function as a phosphodiesterase that inactivates cyclic nucleotides, Pde10a is implicated in the modulation of dopamine signaling, one of the major inputs to MSNs, through dopamine D1 and D2 receptors and the subsequent activation of cAMP production. Studies in rodents indicated that the pharmacological inhibition of Pde10a leads to behavioral effects that phenocopy dopamine D2 receptor inhibition [34]. Moreover, Pde10a knockout mice and patients with biallelic mutation in the *PDE10A* gene exhibit impaired locomotor activity [22,35,36,37]. All these data support a strong implication of Pde10a in striatal function and the regulation of locomotor activity, and thus its possible involvement in the pathophysiology of AHDS. It thus will be interesting to study whether MCT8 patients also present a compromised striatal PDE10A expression and, if so, to what extent PDE10A dysregulation contributes to their pronounced motor problems.

In recent years, a growing number of evidence has been gathered that indeed points to the basal ganglia as an important CNS structure underlying AHDS pathophysiology. MCT8 protein expression was recently found in MSNs in the human striatum, suggesting a direct function of this TH transporter within the basal ganglia circuits [38]. Moreover, MRI studies have detected lesions in the putamen in MCT8 deficiency [39,40]. These findings together with our data further suggest that targeting striatal MSNs may be an alternative therapeutic approach for AHDS aimed at ameliorating the locomotor abnormalities. 

## Data Availability

The mass spectrometry data were deposited in the ProteomeXchange Consortium via the PRIDE partner repository with the dataset identifier PXD045769. A publicly available transcriptomic dataset was used in this study: DOI: 10.1089/thy.2020.0649.

## Supplementary Materials

The following supporting information can be downloaded at: https://www.mdpi.com/article/10.3390/cells12202487/s1, Figure S1: Proteomic profiling of brain sections by LC-MS/MS data. Figure S2: Pvalb interneurons in the striatum. Table S1: List of identified proteins.

## References

[1] Friesema E.C.H., Ganguly S., Abdalla A., Manning Fox J.E., Halestrap A.P., Visser T.J. (2003). Identification of Monocarboxylate Transporter 8 as a Specific Thyroid Hormone Transporter. J. Biol. Chem..

[2] Dumitrescu A.M., Liao X.-H., Best T.B., Brockmann K., Refetoff S. (2004). A Novel Syndrome Combining Thyroid and Neurological Abnormalities Is Associated with Mutations in a Monocarboxylate Transporter Gene. Am. J. Hum. Genet..

[3] Friesema E.C., Grueters A., Biebermann H., Krude H., von Moers A., Reeser M., Barrett T.G., E Mancilla E., Svensson J., Kester M.H. (2004). Association between mutations in a thyroid hormone transporter and severe X-linked psychomotor retardation. Lancet.

[4] Schwartz C.E., May M.M., Carpenter N.J., Rogers R.C., Martin J., Bialer M.G., Ward J., Sanabria J., Marsa S., Lewis J.A. (2005). Allan-Herndon-Dudley Syndrome and the Monocarboxylate Transporter 8 (MCT8) Gene. Am. J. Hum. Genet..

[5] Groeneweg S., van Geest F.S., Peeters R.P., Heuer H., Visser W.E. (2019). Thyroid Hormone Transporters. Endocr. Rev..

[6] Mayerl S., Müller J., Bauer R., Richert S., Kassmann C.M., Darras V.M., Buder K., Boelen A., Visser T.J., Heuer H. (2014). Transporters MCT8 and OATP1C1 maintain murine brain thyroid hormone homeostasis. J. Clin. Investig..

[7] Mayerl S., Chen J., Salveridou E., Boelen A., Darras V.M., Heuer H. (2021). Thyroid Hormone Transporter Deficiency in Mice Impacts Multiple Stages of GABAergic Interneuron Development. Cereb. Cortex.

[8] Reinwald J.R., Weber-Fahr W., Cosa-Linan A., Becker R., Sack M., Falfan-Melgoza C., Gass N., Braun U., von Hohenberg C.C., Chen J. (2022). TRIAC Treatment Improves Impaired Brain Network Function and White Matter Loss in Thyroid Hormone Transporter Mct8/Oatp1c1 Deficient Mice. Int. J. Mol. Sci..

[9] Mayerl S., Heuer H. (2023). Thyroid hormone transporter Mct8/Oatp1c1 deficiency compromises proper oligodendrocyte maturation in the mouse CNS. Neurobiol. Dis..

[10] Espíndola D.L., Morales-Bastos C., Grijota-Martinez C., Liao X.-H., Lev D., Sugo E., Verge C.F., Refetoff S., Bernal J., Guadaño-Ferraz A. (2014). Mutations of the Thyroid Hormone Transporter MCT8 Cause Prenatal Brain Damage and Persistent Hypomyelination. J. Clin. Endocrinol. Metab..

[11] Remerand G., Boespflug-Tanguy O., Tonduti D., Touraine R., Rodriguez D., Curie A., Perreton N., Portes V.D., Sarret C., RMLX/AHDS Study Group (2019). Expanding the phenotypic spectrum of Allan–Herndon–Dudley syndrome in patients with *SLC16A2* mutations. Dev. Med. Child Neurol..

[12] Valcárcel-Hernández V., López-Espíndola D., Guillén-Yunta M., García-Aldea Á., de Toledo Soler I.L., Bárez-López S., Guadaño-Ferraz A. (2021). Deficient thyroid hormone transport to the brain leads to impairments in axonal caliber and oligodendroglial development. Neurobiol. Dis..

[13] Bernal J. (2005). Thyroid Hormones and Brain Development. Vitam. Horm..

[14] Bernal J. (2007). Thyroid hormone receptors in brain development and function. Nat. Clin. Pract. Endocrinol. Metab..

[15] Wallis K., Sjögren M., van Hogerlinden M., Silberberg G., Fisahn A., Nordström K., Larsson L., Westerblad H., de Escobar G.M., Shupliakov O. (2008). Locomotor Deficiencies and Aberrant Development of Subtype-Specific GABAergic Interneurons Caused by an Unliganded Thyroid Hormone Receptor α1. J. Neurosci..

[16] Morte B., Gil-Ibañez P., Heuer H., Bernal J. (2021). Brain Gene Expression in Systemic Hypothyroidism and Mouse Models of MCT8 Deficiency: The Mct8-Oatp1c1-Dio2 Triad. Thyroid®.

[17] Zhang Q., Yang Q., Zhou X., Qin Z., Yi S., Luo J. (2022). Characteristics of Allan-Herndon-Dudley Syndrome in Chinese children: Identification of two novel pathogenic variants of the *SLC16A2* gene. Front. Pediatr..

[18] Heuer H., Schäfer M.K., O’Donnell D., Walker P., Bauer K. (2000). Expression of thyrotropin-releasing hormone receptor 2 (TRH-R2) in the central nervous system of rats. J. Comp. Neurol..

[19] Choi H.M.T., Schwarzkopf M., Fornace M.E., Acharya A., Artavanis G., Stegmaier J., Cunha A., Pierce N.A. (2018). Third-generation *in situ* hybridization chain reaction: Multiplexed, quantitative, sensitive, versatile, robust. Development.

[20] van Geest F.S., Gunhanlar N., Groeneweg S., Visser W.E. (2021). Monocarboxylate Transporter 8 Deficiency: From Pathophysiological Understanding to Therapy Development. Front. Endocrinol..

[21] Martin A.A., Mayerl S. (2023). Local Thyroid Hormone Action in Brain Development. Int. J. Mol. Sci..

[22] Diggle C.P., Rizzo S.J.S., Popiolek M., Hinttala R., Schülke J.-P., Kurian M.A., Carr I.M., Markham A.F., Bonthron D.T., Watson C. (2016). Biallelic Mutations in PDE10A Lead to Loss of Striatal PDE10A and a Hyperkinetic Movement Disorder with Onset in Infancy. Am. J. Hum. Genet..

[23] Masnada S., Sarret C., Antonello C.E., Fadilah A., Krude H., Mura E., Mordekar S., Nicita F., Olivotto S., Orcesi S. (2021). Movement disorders in MCT8 deficiency/Allan-Herndon-Dudley Syndrome. Mol. Genet. Metab..

[24] Vancamp P., Demeneix B.A., Remaud S. (2020). Monocarboxylate Transporter 8 Deficiency: Delayed or Permanent Hypomyelination?. Front. Endocrinol..

[25] Bárez-López S., Grijota-Martínez C., Ausó E., Frutos M.F.-D., Montero-Pedrazuela A., Guadaño-Ferraz A. (2019). Adult Mice Lacking Mct8 and Dio2 Proteins Present Alterations in Peripheral Thyroid Hormone Levels and Severe Brain and Motor Skill Impairments. Thyroid®.

[26] Zekri Y., Guyot R., Flamant F. (2022). An Atlas of Thyroid Hormone Receptors’ Target Genes in Mouse Tissues. Int. J. Mol. Sci..

[27] Frei J.A., Andermatt I., Gesemann M., Stoeckli E.T. (2014). The synaptic cell adhesion molecules SynCAMs are involved in sensory axon pathfinding by regulating axon-axon contacts. J. Cell Sci..

[28] Airola M.V., Hannun Y.A. (2013). Sphingolipid Metabolism and Neutral Sphingomyelinases. Sphingolipids: Basic Science and Drug Development.

[29] Diez D., Grijota-Martinez C., Agretti P., De Marco G., Tonacchera M., Pinchera A., de Escobar G.M., Bernal J., Morte B. (2008). Thyroid Hormone Action in the Adult Brain: Gene Expression Profiling of the Effects of Single and Multiple Doses of Triiodo-l-Thyronine in the Rat Striatum. Endocrinology.

[30] Conti M., Beavo J. (2007). Biochemistry and Physiology of Cyclic Nucleotide Phosphodiesterases: Essential Components in Cyclic Nucleotide Signaling. Annu. Rev. Biochem..

[31] Albin R.L., Young A.B., Penney J.B. (1989). The functional anatomy of basal ganglia disorders. Trends Neurosci..

[32] Haber S.N., Fudge J.L., McFarland N.R. (2000). Striatonigrostriatal Pathways in Primates Form an Ascending Spiral from the Shell to the Dorsolateral Striatum. J. Neurosci..

[33] Haber S.N. (2016). Corticostriatal circuitry. Dialog-Clin. Neurosci..

[34] Menniti F.S., Chappie T.A., Schmidt C.J. (2021). PDE10A Inhibitors—Clinical Failure or Window into Antipsychotic Drug Action?. Front. Neurosci..

[35] Mencacci N.E., Kamsteeg E.-J., Nakashima K., R’bibo L., Lynch D.S., Balint B., Willemsen M.A., Adams M.E., Wiethoff S., Suzuki K. (2016). De Novo Mutations in PDE10A Cause Childhood-Onset Chorea with Bilateral Striatal Lesions. Am. J. Hum. Genet..

[36] Trieschmann G., Wach K., Berweck S., Zech M., Abel M., Tilgner E. (2022). A Novel Homozygous PDE 10A Variant Leading to Infantile-Onset Hyperkinesia. Neuropediatrics.

[37] Siuciak J.A., McCarthy S.A., Chapin D.S., Martin A.N., Harms J.F., Schmidt C.J. (2008). Behavioral characterization of mice deficient in the phosphodiesterase-10A (PDE10A) enzyme on a C57/Bl6N congenic background. Neuropharmacology.

[38] Wang T., Wang Y., Montero-Pedrazuela A., Prensa L., Guadaño-Ferraz A., Rausell E. (2023). Thyroid Hormone Transporters MCT8 and OATP1C1 Are Expressed in Projection Neurons and Interneurons of Basal Ganglia and Motor Thalamus in the Adult Human and Macaque Brains. Int. J. Mol. Sci..

[39] Kakinuma H., Itoh M., Takahashi H. (2005). A Novel Mutation in the Monocarboxylate Transporter 8 Gene in a Boy with Putamen Lesions and Low Free T4 Levels in Cerebrospinal Fluid. J. Pediatr..

[40] Tonduti D., Vanderver A., Berardinelli A., Schmidt J.L., Collins C.D., Novara F., Di Genni A., Mita A., Triulzi F., Brunstrom-Hernandez J.E. (2012). MCT8 Deficiency. J. Child Neurol..

