# Supplementary material for: Proteome Analysis of Thyroid Hormone Transporter Mct8/Oatp1c1-Deficient Mice Reveals Novel Dysregulated Target Molecules Involved in Locomotor Function
Source: Cells. 2023 Oct 19;12(20):2487. doi: 10.3390/cells12202487 (PMC10605308; doi:10.3390/cells12202487)
Supplement: Supplementary file 1 [file cells-12-02487-s001.zip › cells-2663733-supplementary.pdf]

Supplementary Materials

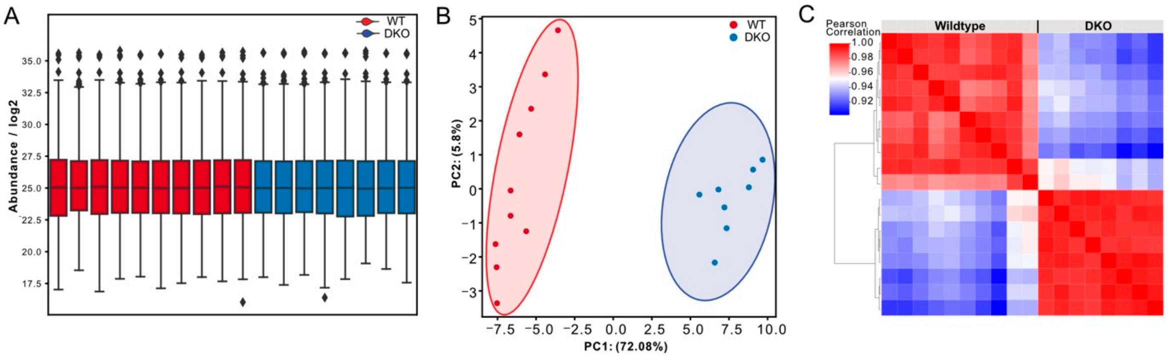

Supplementary Figure S1. Proteomic profiling of brain sections by LC-MS/MS data. (**A**) Box plot illustrating protein abundance distributions of all samples after loess normalization and imputation. (**B**) Principal component (PC) analysis and (**C**) pearson correlation clustermap with proteins filtered by q <0.01 indicate clear clustering of the samples of both conditions with no outlier samples.

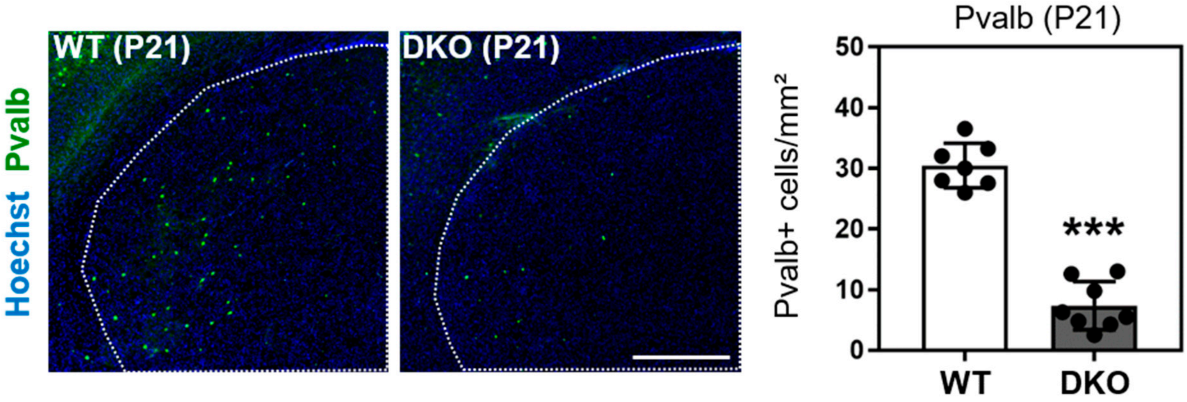

Supplementary Figure S2. Pvalb interneurons in the striatum. Immunofluorescent staining of Pvalb protein (in green) and enumeration of Pvalb-expressing cells demonstrating reduced values in DKO mice at P21. Hoechst33258-counterstained cell nuclei appear blue. Scale bar: 500 µm. n = 7–8. ***, *p* < 0.001.
